# Supplementary material for: Is there an impact of public smoking bans on self-reported smoking status and exposure to secondhand smoke?
Source: BMC Public Health. 2011 Mar 3;11:146. doi: 10.1186/1471-2458-11-146 (PMC3064640; doi:10.1186/1471-2458-11-146)
Supplement: Additional file 1 — CCHS survey questions pertaining to secondhand smoke and smoking status. Survey questions from the 2.1 and 3.1 versions of the CCHS that relate to secondhand exposure in different settings and current smoking status. [file 1471-2458-11-146-S1.DOC]

Additional file 1: CCHS survey questions pertaining to secondhand smoke and smoking status:

|  | Respondents were asked the following questions: |
| --- | --- |
| Exposure to SHS in public places | Respondents reported being exposed to secondhand smoke in public places on every day or almost every day in the past month |
| Smoking restriction at work | Employed population aged 15 to 75 who reported that smoking is completely restricted at their place of work. |
| Smoking restriction at home | Respondents reported that smokers are asked to refrain from smoking in the house |
| Exposure to secondhand smoke in vehicles | Non-smoking population aged 12 and over who reported being exposed to second-hand smoke in private vehicles on every day or almost every day in the past month. |
| Current Smoking | Respondents reported being a current smoker. This includes daily smokers (identified smoking cigarettes every day and occasional smokers who reported smoking cigarettes occasionally. |
